# First assessment of the performance of an implantable continuous glucose monitoring system through 180 days in a primarily adolescent population with type 1 diabetes

**DOI:** 10.1111/dom.13726

**Published:** 2019-04-23

**Authors:** Ronnie Aronson, Alexander Abitbol, Katherine S. Tweden

**Affiliations:** ^1^ LMC Diabetes and Endocrinology Toronto Ontario Canada; ^2^ Senseonics, Incorporated Germantown Maryland USA

**Keywords:** clinical trial, continuous glucose monitoring, type 1 diabetes

## Abstract

**Aim:**

To investigate the performance of the Eversense XL implantable continuous glucose monitoring (CGM) system through 180 days in a primarily adolescent population with type 1 diabetes (T1D).

**Materials and methods:**

This prospective, single‐centre, single‐arm, 180‐day study evaluated the effectiveness and safety of the implantable CGM system in Canadian adolescent and adult subjects with T1D. Accuracy measures included mean absolute relative difference (MARD), 15/15% agreement between CGM glucose and blood glucose measured by Yellow Springs Instruments and surveillance error grid analysis. Adolescent subjects received one sensor in the upper arm and adult subjects received one sensor in each upper arm. In‐clinic CGM system accuracy studies were performed every 30 days. The safety assessment included the incidence of adverse events related to either device or the insertion/removal procedure through 180 days.

**Results:**

Thirty‐six subjects (30 adolescent/6 adult, 13 female/23 male, mean age 17 ± 9.2 years, mean body mass index 22 ± 4 kg/m^2^) received the CGM system. Overall MARD was 9.4% (95% CI: 8.6%‐10.5%). CGM system agreement at 15/15% (N = 7163) through 60, 120 and 180 days was 82.9% (95% CI: 78.4%‐86.1%), 83.6% (95% CI: 80.4%‐85.7%) and 83.4% (95% CI: 79.7%‐85.5%), respectively. Surveillance error grid analysis showed 98.4% of paired values in clinically acceptable error zones A and B. No insertion/removal or device‐related serious adverse events were reported.

**Conclusion:**

The Eversense XL CGM system is safe and accurate through 180 days in a primarily adolescent population of subjects with T1D.

## 1 INTRODUCTION

People living with diabetes have traditionally relied upon multiple daily self‐monitoring of blood glucose (SMBG) via capillary fingertip glucose to guide their diabetes self‐care decisions. The known limitations of SMBG include patient engagement as well as the limited window of insight into glucose control and the lack of detailed trend information. Real time continuous glucose monitoring (CGM) systems increase the amount of actionable information available to the patient and alerts patients to trends outside the ideal range using predictive alerts before hypoglycaemic or hyperglycaemic conditions occur.

Despite the advantages of CGM systems, their adoption has been slow to grow, increasing from only 9% in 2014 to 38% in 2018.^1^ Their use is even lower among individuals with type 2 diabetes. One of the key barriers to long‐term adoption of CGM technology has been tiredness of use, with patients reporting discontinuation in the first year because of many reasons including perceived inaccuracy (53%), wear discomfort (47%), insertion pain (31%) and skin irritation (41%).^2^

In contrast to the documented benefits of CGM use in adult patients with diabetes,^3^, ^4^ studies specifically involving paediatric patients have shown more inconsistent findings.^5^ This disparity in benefits may be partly as a result of even more inconsistent use than that observed in adults. In the JDRF CGM 6‐month study, among young adults (aged 15‐24 years), only 30% wore a CGM device ≥6 days per week, in contrast to 86% of subjects ≥25 years of age.^5^ Barriers to adoption by this age group have been attributed to poor accuracy of the CGMs available at the time^6^ and the nuisance of frequent alerts and alarms.^7^

Traditional enzyme‐based CGM systems last 7 to 14 days and require a patient to self‐insert a transcutaneous sensor. Once inserted, the sensor is secured with adhesive patches designed to last the 7‐to‐14 day sensor lifespan. The Eversense XL CGM (Senseonics Incorporated, Germantown, Maryland, USA) sensor technology is based on fluorescence that is designed to last an extended period and uses a sensor that is fully inserted into the subcutaneous tissue on the upper arm, a smart transmitter worn externally over the sensor, and a mobile app that displays glucose information on a handheld device. An abiotic (non‐enzyme) fluorescent glucose‐indicating polymer is grafted to the poly(methyl methacrylate) shell of the sensor. A dexamethasone‐eluting silicone rubber collar is also attached to the outside of the sensor. Glucose binding leads to increased fluorescence intensity, which is measured by the sensor optical components. The overlying wearable transmitter powers the sensor through an inductive link, receives the fluorescence intensity data and converts it into glucose data, which is then transmitted via Bluetooth for display on the mobile app.

The PRECISE study^8^ assessed the original Eversense CGM system in 71 adults with type 1 diabetes (T1D) or type 2 diabetes (T2D) over 180 days and found a mean absolute relative difference (MARD) of 11.6% and 84% of CGM values within 15 mg/dL or 20% of the Yellow Springs Instruments (YSI) reference glucose values (transition at 75 mg/dL). Median sensor lifespan was 149 days (IQR 97‐180) with 82% functional through day 90 and 40% functional through day 180. The subsequent system generation included repositioning of the dexamethasone collar and updated software, and was studied in 90 adults with T1D and T2D through 90 days with a resultant MARD of 8.8% [for blood glucose (BG) 40 to 400 mg/dL] and a 15/15% metric of 86%.^9^ Ninety‐one per cent of sensors were functional through day 90.

The full lifespan of the new CGM system configuration has not been fully assessed and its efficacy in a paediatric population has not yet been evaluated. This study therefore undertook the investigation of safety and effectiveness of the implantable CGM system in a primarily adolescent population with T1D for up to 180 days.

## 2 MATERIALS AND METHODS

### 2.1 Study design

The study used a non‐randomized, non‐blinded, prospective, single‐arm, single‐centre design. Subjects were ≥12 years of age and had T1D for ≥1 year. Patients were excluded if they had any condition that might have prevented the placement or removal of the sensor, any condition that might require an MRI, or any other active implanted device.

Following an initial screening visit, subjects returned for sensor insertion followed by seven sensor accuracy assessment visits at 30 day intervals. Each sensor was inserted subcutaneously in the upper arm through a small (5‐6 mm) incision which was subsequently closed with Steri‐Strips. Subjects ≥18 years of age had two sensors inserted, with one designated as an unblinded (primary) sensor and the other as a blinded (secondary) sensor. Subjects <18 years of age had one sensor inserted which was unblinded. Subjects were issued an iPod to display glucose measurements from the unblinded sensor. Subjects were instructed to wear the transmitter over the sensor(s) for data collection, glucose display and physical glucose alerts (except during transmitter charging, bathing or water activity). Subjects were provided with a BG meter (CONTOUR NEXT USB, Ascensia Diabetes Care, Parsippany, New Jersey, USA) for use throughout the study, and were asked to perform calibration twice daily. During home use, all diabetes management decisions were to be based on BG monitoring rather than CGM values.

For the sensor accuracy visits, patients were admitted for 5‐7.5 hours, consumed their typical diet and followed their own self‐managed treatment regimen. No manipulation of glucose levels was performed. Venous samples (Yellow Springs Instruments, Yellow Springs, Ohio, USA) were drawn every 15 minutes for blood glucose levels ≥75 mg/dl (4.2 mmol/L) and every 5 minutes for blood glucose levels <75 mg/dl (4.2 mmol/L). Each CGM measurement was paired to the corresponding YSI glucose measurement obtained within 5 minutes of the CGM value. HbA1c levels were also assessed at day 90 and day 180. A custom‐designed patient satisfaction questionnaire was administered at day 30 and after sensor removal.

Following the final accuracy assessment at day 180, the sensors were removed. Ten days following removal (day 190), subjects returned for follow‐up and the removal site was inspected.

### 2.2 Outcome measures

The primary effectiveness endpoint was MARD for all paired sensor and reference measurements through 180 days postinsertion. The primary safety endpoint was the incidence of device‐related or insertion/removal procedure‐related serious adverse events (SAEs) in the clinic and during home use through 180 days postinsertion.

Secondary outcomes included surveillance and consensus error grid analyses, Bland‐Altman analysis, transmitter wear time, alert performance and sensor longevity. Additional secondary outcomes included monthly analysis of CGM system agreement with YSI and assessment of performance in hypo‐, eu‐ and hyperglycaemic regions (≤70, 71‐180 and >180 mg/dL, respectively).

Additional safety outcomes included insertion/removal procedure adverse events (AEs) and device‐related AEs, hospitalization as a result of hypoglycaemia, hyperglycaemia or ketoacidosis, and incidence of hypoglycaemic and hyperglycaemic events occurring during home use.

### 2.3 Statistical methods

To detect a difference of 4% in MARD with 80% power and a one‐sided significance level of 0.0250, a total of 99 independent paired glucose values are required based on one‐sample t‐test. Following adjustments accounting for within‐subject correlation and non‐normality of ARD data, 1286 data pairs were required. At the expected 205 pairs per patient, eight enrolled subjects would be required. To ensure a robust cross‐section of the target population, over the full 180‐day evaluation period, and to accommodate subject dropouts over time, a target number of 36 evaluable subjects was determined. Because the sensor was not involved in subject management, the cohort expansion did not pose an additional safety concern.

The prespecified analysis population for the effectiveness endpoints was based on all evaluable glucose data from all subjects with at least one paired glucose reading. All other effectiveness analyses were evaluated using descriptive statistics. Sensor longevity was defined as the number of days between the implant and the last day the sensor remained functional, and was assessed by time‐to‐event analyses. Sensors which remained functional through 180 days were censored at day 180. All subjects who had a sensor placed were included in the safety analysis population.

The Kaplan‐Meier method^10^ was used to estimate the probability of sensor survival through 180 days, and the log‐log method was used to generate 95% confidence intervals for the survival probabilities.^11^

Subjects in the study included adolescents and adults. While age was not expected to have an impact on device longevity, an exploratory analysis using a Cox proportional hazards model evaluated the impact of age on device longevity. MATLAB (MathWorks, Natick, Massachusetts, USA) and R version 3.4.1 (https://www.r-project.org/about.html) were used for all statistical analyses.

The study (www.clinicaltrials.gov: NCT02933164) was performed in accordance with the Declaration of Helsinki and was approved by an ethics committee. Both written and verbal informed consent were obtained from all study subjects.

## 3 RESULTS

A total of 36 subjects (n = 30 children; n = 6 adults) were enrolled in the study (Table 1). Forty‐three sensors were placed in the study (30 single sensor subjects, 12 bilateral dual sensor subjects and one subject who received one replacement sensor because of a suspected technical device failure). Twenty‐eight subjects (78%) completed the study with day‐180 data collection. Eight (22%) subjects experienced a sensor replacement alert prior to day 180 which ended glucose data collection. One subject withdrew consent because of an inability to tolerate intravenous access for in‐clinic accuracy testing.

**Table 1:** Baseline characteristics of study subjects

Characteristic	Paediatric cohort(N = 30)	Adult cohort(N = 6)	Total(N = 36)
Age, mean (SD)	13.9 (1.4)	32.0 (15.9)	16.9 (9.2)
Male sex, n (%)	20 (67%)	3 (50%)	23 (64%)
Caucasian race, n (%)	29 (97%)	6 (100%)	35 (97%)
Hispanic ethnicity, n (%)	1 (3%)	0 (0%)	1 (3%)
HbA1c (%)	8.1 (1.5)	7.5 (1.0)	8.0 (1.4)
HbA1c (mmol/mol)	65 (16.4)	58 (10.9)	64 (15.3)
Body mass index (kg/m^2^), mean (SD)	21.7 (3.6)	24.8 (4.2)	22.3 (3.8)
Continuous insulin infusion pump	26 (87%)	6 (100%)	32 (89%)
Multiple daily injections	4 (13%)	0 (0%)	4 (11%)
Duration of diabetes (years), mean (SD)	6.0 (3.8)	19.8 (15.5)	8.3 (8.6)
Prior continuous glucose monitoring use, n (%)	17 (57%)	6 (100%)	23 (64%)

Of the subjects, 30 (83%) were youths aged 12‐17 [mean (SD): 13.9 (1.4)] years and with diabetes duration of 6.0 (3.8) years. There were 6 (17%) adults with a mean age of 32.0 (15.9) and diabetes duration of 19.8 (15.5) years. Among the children, 26 (87%) were using insulin pump therapy and 17 (57%) had previously used CGM. In the adult group, all were using insulin pump therapy and all had used CGM previously. Table 1 provides a summary of demographics and baseline characteristics of the study subjects.

### 3.1 Effectiveness outcomes

There were a total of 7163 matched pairs of CGM and YSI glucose readings. The primary outcome of MARD across 40 to 400 mg/dL, over the 180‐day duration, was 9.4% (95% CI: 8.6%‐10.5%) in the total cohort and 9.7% in the paediatric cohort (95% CI: 8.6%‐10.8%). MARD on day 1 was higher at 13.3 (13%), improving to 10.6 (10.7%) by day 30 and then ranging between 9.1% and 9.7% over the full 180‐day duration (Table A1). CGM system agreement with YSI glucose within 15 mg/dL (<100 mg/dL) or 15% of YSI glucose values, through 60, 120 and 180 days, was 82.9%, 83.6% and 83.4%, respectively. MARD and system agreement in each successive monthly interval are shown in Table 2.

**Table 2:** Continuous glucose monitoring (CGM) system agreement with Yellow Springs Instruments (YSI) glucose within YSI glucose range 40‐400 mg/dL

	Total cohort			Paediatric cohort		
Duration	Number of paired CGM and YSI reference	MARD (%) (SD)	Per cent within 15/15% reference	Number of paired CGM and YSI reference	MARD (%) (SD)	Per cent within 15/15% reference
Day 1‐30	2017	10.6 (10.7)	79.6%	1522	10.6 (11.4)	79.8
Day 31‐60	1164	7.9 (6.9)	88.7%	876	7.7 (7.0)	89.3
Day 61‐90	1071	7.7 (8.3)	88.2%	840	8.0 (8.7)	87.6
Day 91‐120	1116	9.7 (9.3)	81.2%	919	10.4 (9.9)	77.8
Day 121‐150	896	9.7 (9.6)	83.6%	689	10.4 (10.5)	81.9
Day 151‐180	899	10.1 (12.3)	82.2%	717	10.6 (13.4)	80.3

Abbreviations: MARD, mean absolute relative difference; SD, standard deviation.

Over the first 90 days, MARD across glycaemic ranges (Table 3) was 9.6% (9.2) in euglycaemia (71‐180 mg/dL), 10.5% (8.0) in hypoglycaemia (≤70 mg/dL) and 6.7% (7.6) in hyperglycaemia (>180 mg/dL). Within each range, MARD was generally higher in the paediatric subgroup versus the adult subgroup, but the differences were not statistically significant (Table 3).

**Table 3:** Mean absolute relative difference (MARD) by glycaemic range (first 90 days)

	Full cohort		Paediatric only		Adult only	
YSI glucose range	MARD (%) (SD)	No. of paired points	MARD (%) (SD)	No. of paired points	MARD (%) (SD)	No. of paired points
<70	10.5 (8.0)	148 (18)	10.6 (8.1)	109 (14)	10.3 (7.6)	39 (4)
70‐180	9.6 (9.2)	2928 (35)	9.8 (9.7)	2090 (30)	9.1 (7.4)	838 (5)
>180	6.7 (7.6)	1201 (35)	6.8 (7.8)	1064 (30)	6.6 (6.3)	137 (5)
Overall	9.1 (9.2)	4277 (35)	9.1 (9.6)	3263 (30)	9.1 (7.7)	1014 (5)

Abbreviations: SD, standard deviation; YSI, Yellow Springs Instruments.

**Figure 1:**
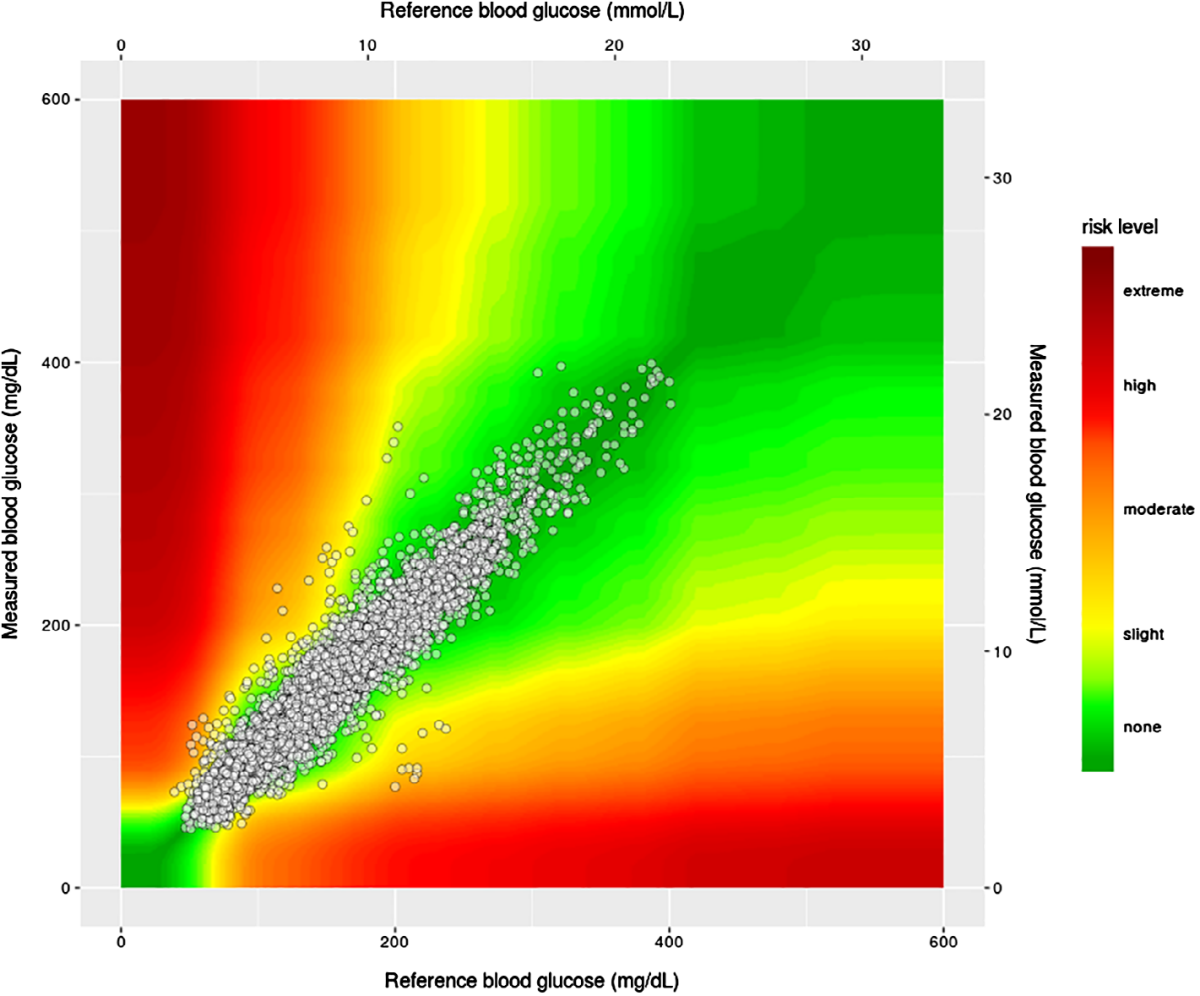
Surveillance error grid analysis for sensor glucose versus reference plasma glucose (Yellow Springs Instruments)

The surveillance error grid showed 98.4% within the green zone (Figure 1), indicating no clinical risk for errors in assessment of hypo‐ and hyperglycaemia. Consensus error grid analysis similarly found 99.6% of pairs in the clinically acceptable error zones of A (93.4%) and B (6.2%) (Figure S1, see the supporting information for this article).

The Bland‐Altman analysis (Figure S2) showed a non‐significant bias of 2.5 mg/dL (95% CI: ‐32.5, 37.5) in CGM glucose compared with YSI glucose.

### 3.2 Safety Outcomes

In the paediatric group, HbA1c was 8.1% ± 1.5% at baseline and slightly lower at 90 days (7.5% ± 1.1%, 58 ± 12.0 mmol/mol) and 180 days (7.9% ± 1.4%, 63 ± 15.3 mmol/mol). The adult group showed a stable HbA1c of 7.5% ± 1.0% at baseline, 7.4% ± 0.8% (57 ± 8.7 mmol/mol) at 90 days and 7.6% ± 0.9% (60 ± 9.8 mmol/mol) at 180 days.

A total of 37 sensors implanted in 30 subjects were included in the analysis. At postimplant days 90, 120, 150 and 180, the estimated probabilities of sensor survival were 97%, 94%, 81% and 78%, respectively (Figure S3).

Sensors were removed prior to 180 days for the following reasons: withdrawal of consent 1 day after insertion because of difficulty with the blood collection protocol (1); inability to maintain connection with any transmitter (2); and sensor replacement alarms at day 136 (1), day 142 (1), day 146 (3) and day 165 (1).

An exploratory Cox proportional hazards regression analysis found that subject age was not associated with sensor longevity (hazard ratio = 1.02, 95% CI = 0.94‐1.10; *P* = 0.68).

The sensor insertion and removal procedures were well tolerated with no SAEs. The most common AEs related to the insertion/removal process were presyncope (2), nausea (2) and vomiting (2). Sensor fracture occurred in the process of removal in two subjects without further impact.

The implanted sensor and wearable transmitter combination were also well tolerated with a median wear time of 23 ± 6.6 hours daily in the total cohort, and was not different in the paediatric cohort (23 ± 6.1 hours). Skin reactions to the sensor were mild when they occurred and included skin thinning (13), discoloration (2) and bruise (2). One subject experienced a skin reaction to the adhesive patch which was moderate and followed a prior history of chronic dermatitis and prior intolerance of other adhesives. All skin changes resolved within 1‐24 weeks of sensor removal. There were no infections.

Subjects completed two questionnaires (Tables A2 and A3) developed to assess the subjects’ perceived impact of the CGM system and its appeal. The proportion of subjects who agreed or highly agreed that the CGM was easy to use was 82%; 90% felt that the mobile application for viewing glucose and trends was easy to use; and 87% felt confident in the alarm's reliability to warn of glucose extremes. Regarding comfort at the sensor/transmitter site, 76% of subjects agreed or highly agreed that they had not experienced pain or discomfort when using the sensor. Ninety per cent of subjects liked the ability to see their glucose on their iPod and 82% reported actively using the glucose display at least every other hour (Table A4). Finally, 78% liked the longer sensor duration.

## 4 DISCUSSION

The results of this open‐label study show that the new Eversense XL system is accurate and safe in a primarily adolescent population over the 180‐day sensor life with a MARD of 9.4% and 83.4% of CGM glucose readings within 15/15% of the reference glucose values. This study also confirmed clinically meaningful accuracy throughout the sensor lifespan. The surveillance error grid showed 98.4% in the green zone and the consensus error grid showed 99.6% of values in zones A and B. The device was well tolerated with generally mild skin‐related AEs that resolved without intervention after sensor removal. The insertion and removal procedures were also well tolerated with mild, short‐duration AEs in seven subjects. Importantly, no infections and no related SAEs were observed. In addition, the study showed that the reconfiguration of the sensor in the new Eversense XL system provided greater longevity than the original configuration with 97% functional at day 90 and 78% functional at day 180. Study subjects favourably rated the long duration sensor and consistently wore the transmitter 96% of the time (23 hours/day), showing that an implantable sensor is consistent with an adolescent lifestyle. The questionnaire results also showed that the subjects felt the CGM was easy to use (82%) and that the app was easy to use (90%). Importantly, almost 90% of the subjects felt confident in the alarm's reliability to warn of BG extremes, and the majority (78%) liked the longevity of the sensor. Finally, although impact on glycaemia was not a defined outcome, there was a downward trend in mean HbA1c results in the adolescent cohort from baseline to 90 and 180 days. The baseline HbA1c of this adolescent cohort (8.1%) was generally lower than recently reported for adolescents with T1D (9.3%).^1^

Although CGM use by all age groups has been increasing since 2011, it remains lowest among adolescents, at 16% in the European Prospective Diabetes Follow‐up Registry^12^ and at 24% of participants in the most recent report from T1D Exchange.^1^ Even among active users, median duration of wear in children and adolescents has lagged behind adult usage in the past (23 days and 21 days vs. 29 days of the prior month, respectively).^13^ Similarly, the benefit of CGM use and its association with improved HbA1c was highly significant among adults and less so with children and adolescents. As many as 41% of CGM users abandoned their device within their first year^1^ and subsequent surveys have suggested that there are age‐related differences in perceptions of barriers within the T1D Exchange cohort.^14^ Younger adult age groups identified more barriers to CGM use, more reasons for discontinuing them, and reported higher levels of diabetes distress generally. Although all age groups most frequently complained about the ‘hassle of wearing devices’, the younger age groups more frequently identified additional barriers such as not liking both ‘diabetes devices on my body’ and ‘how diabetes devices look on my body’. Younger age groups also described more anxiety about device reliability and what others will think (17.7%) and notice (16.9%).^14^ Among adolescents especially, the use of CGM may alter self‐care behaviour to unexpected extremes, based on the unique dynamics of desire for autonomy, distrust of technology and vulnerability to risk‐taking behaviour.^15^

Our study had several limitations. Although we were able to study the CGM system in a meaningful number of adolescents, we were only able to include a small number of adults. We evaluated accuracy versus plasma glucose with multiple paired sample testing in a clinic setting but we did not manipulate plasm glucose levels to extremes, nor did we ‘clamp’ plasma glucose levels at specific settings. We were able to assess subjects’ experience with detailed questionnaires but we did not use currently validated tools. Finally, although the skin adverse changes that occurred were mild, subjects were not assessed by a dermatologist to better define these changes.

In conclusion, the Eversense XL CGM system is safe and accurate through 180 days of sensor wear in a primarily adolescent population. Children and adolescents face the challenge of transition to adult medical care, as well as the burden of navigating unique personal and social challenges. These challenges are reflected in their reported resistance to using devices such as CGM, in particular to wearing the device, how it will look on their body and what others will think. The high rate of daily wear and the favourable survey responses reported in this cohort suggest that the implanted Eversense sensor may be promising in its ability to potentially overcome these unique paediatric challenges. Future studies should address additional questions regarding accuracy in a larger population, in both free‐living settings and in the clinic, during controlled extremes of glycaemia and in response to glucose load and to exercise.

## CONFLICT OF INTEREST

R.A. reports research support from AstraZeneca, Eli Lilly, Boehringer Ingelheim, Valeant, Janssen and Senseonics, and has been part of an advisory panel for Novo Nordisk and Sanofi.

A.A. reports research support or honoraria from Amgen, AstraZeneca, Boehringer Ingelheim, Eli Lilly, Gilead, GlaskoSmithKline, JA DeSeve, JDRF, Lexicon, Merck Canada, Novo Nordisk, Pfizer, Sanofi, Senseonics, Zealand and Xeris.

K.S.T. is an employee of Senseonics, Inc.

## AUTHOR CONTRIBUTIONS

R.A. and K.S.T. designed the study and wrote the first draft of the manuscript, and A.A. provided critical revisions to the manuscript. All authors edited and approved the final version of the manuscript. Ronnie Aronson is the guarantor of this article and takes responsibility for the contents of the article.

## Supporting information


**Figure S1.** Consensus Error Grid analysis for sensor glucose versus reference plasma glucose (YSI)

**Figure S2.** Bland‐Altman plot of the agreement between sensor glucose versus reference plasma glucose (YSI)

**Figure S3.** Kaplan‐Meier analysis of sensor survival over time
