# Supplementary figures and images for: First assessment of the performance of an implantable continuous glucose monitoring system through 180 days in a primarily adolescent population with type 1 diabetes
Source: Diabetes Obes Metab. 2019 Apr 23;21(7):1689–94. doi: 10.1111/dom.13726 (PMC6618327; doi:10.1111/dom.13726)

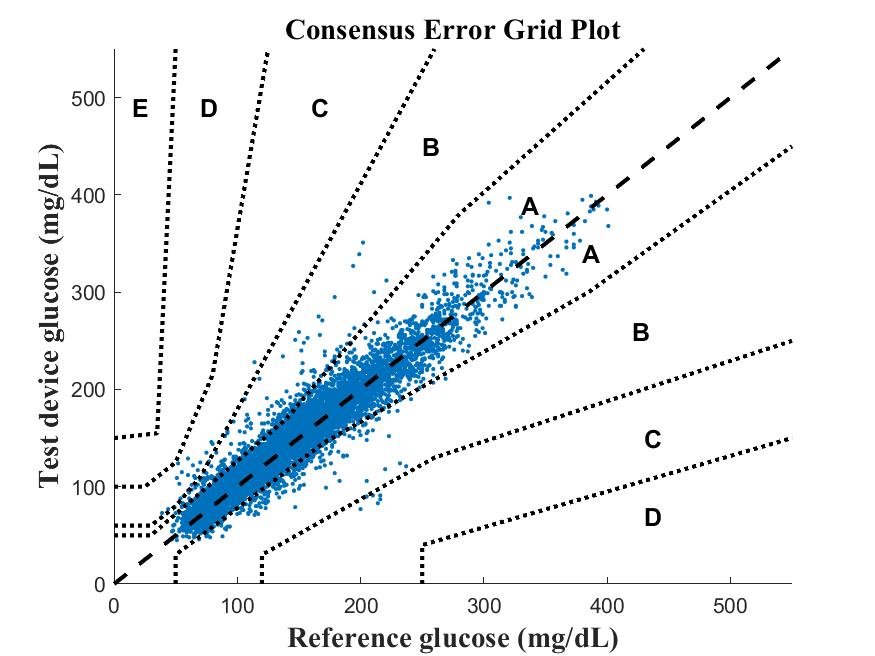

Supplement: Supplementary file 1 — Figure S1. Consensus Error Grid analysis for sensor glucose versus reference plasma glucose (YSI) [file DOM-21-1689-s001.tif]

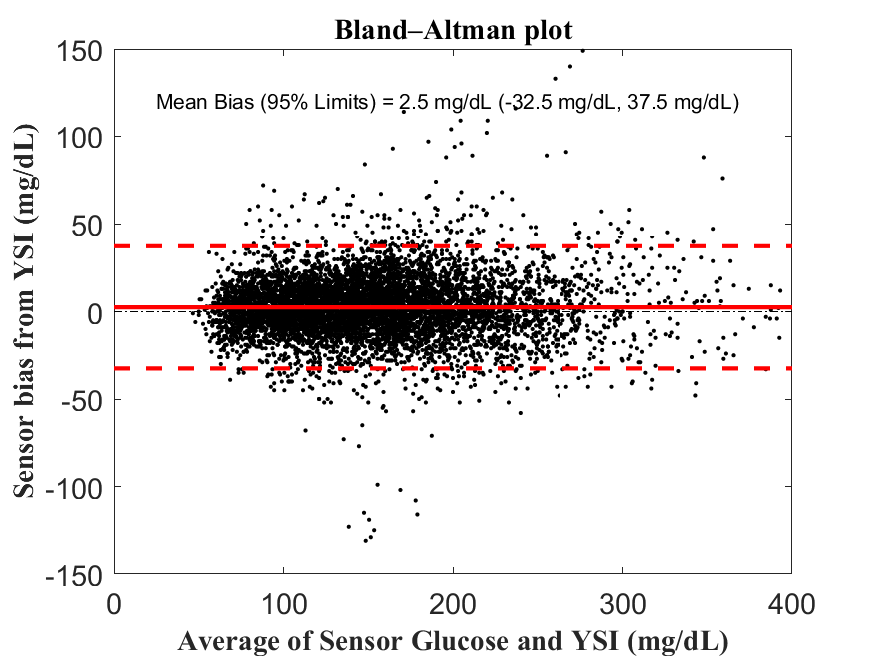

Supplement: Supplementary file 2 — Figure S2. Bland‐Altman plot of the agreement between sensor glucose versus reference plasma glucose (YSI) [file DOM-21-1689-s002.tif]

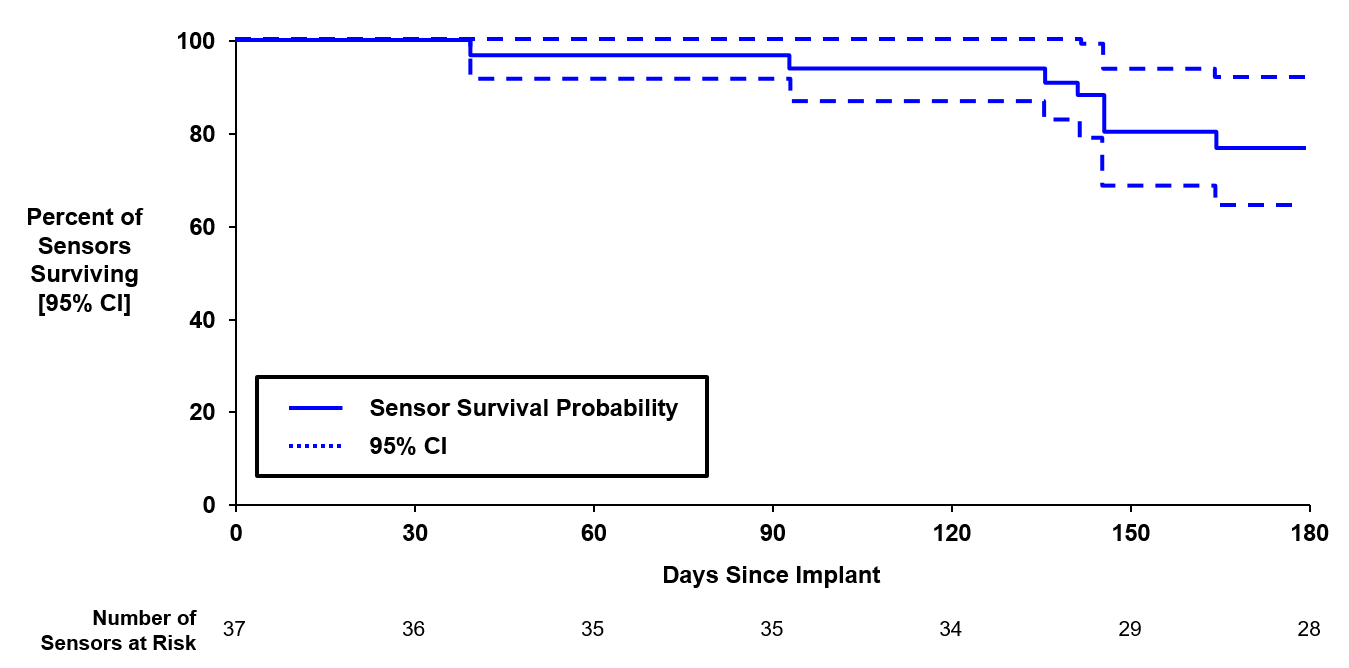

Supplement: Supplementary file 3 — Figure S3. Kaplan‐Meier analysis of sensor survival over time [file DOM-21-1689-s003.tif]
